# Not another round: microtubule-associated proteins and the control of fruit shape in tomato

**DOI:** 10.1093/plcell/koad238

**Published:** 2023-09-14

**Authors:** Humberto Herrera-Ubaldo

**Affiliations:** Assistant Features Editor, The Plant Cell, American Society of Plant Biologists; Department of Plant Sciences, University of Cambridge, Cambridge CB2 3EA, UK

Fruits with extravagant shapes are attractive to consumers. Buddha-shaped pears or Japanese square-shaped watermelons are extreme examples that are produced by restricting fruit growth during development. At the molecular level, the final shape of a fruit is determined partly by the number of locules through the regulation of genes controlling carpel initiation (e.g. *WUSCHEL*, *FASCIATED*, *LOCULE NUMBER*). Fruit shape also relies on controlled cell division and expansion patterns regulated by, for example, *OVATE FAMILY PROTEIN 20* or *SUN1* (van der Knaap et al. 2014). SUN1, which is particularly important for fruit expansion, localizes to the microtubules and affects microtubule organization and cell division in several species (Xiao et al. 2008; Bürstenbinder et al. 2017). Microtubule-associated proteins (MAPs) regulate cell growth and processes related to morphogenesis. However, it is not clear how MAPs regulate cell morphology and organ shape during reproductive development. In this issue, **Zhiru Bao and colleagues** (Bao et al. 2023) report the identification of MAP70-1, MAP70-2, and the IQ67-domain protein IQD21a as regulators of fruit shape that orchestrate microtubule dynamics involved in defining long, round, or flat tomato fruits.

The authors studied the MAP70 proteins in tomato (*Solanum lycopersicum*), a group of 5 plant-specific, microtubule-related proteins that localize to microtubules; SlMAP70-1 was chosen for further analysis since it has a higher expression during fruit development. The overexpression of *SlMAP70-1* generated elongated fruits, while the knock-down lines generated short and flat ones (see Fig.). The *slmap70-1* and *slmap70-2* knock-out lines resulted in short fruits, and fruits were even shorter in the double mutant *slmap70-1/2*. At the cellular level, the SlMAP70 lines displayed contrasting features of microtubule organization. The *SlMAP70* overexpression lines showed reduced cell circularity values (complex shapes), while the cells were more circular in the knock-down lines. Although the lobe number of the cells was not affected, the lobe length was shorter in the knock-down or knock-out lines and longer in the overexpression lines.

**Figure.**
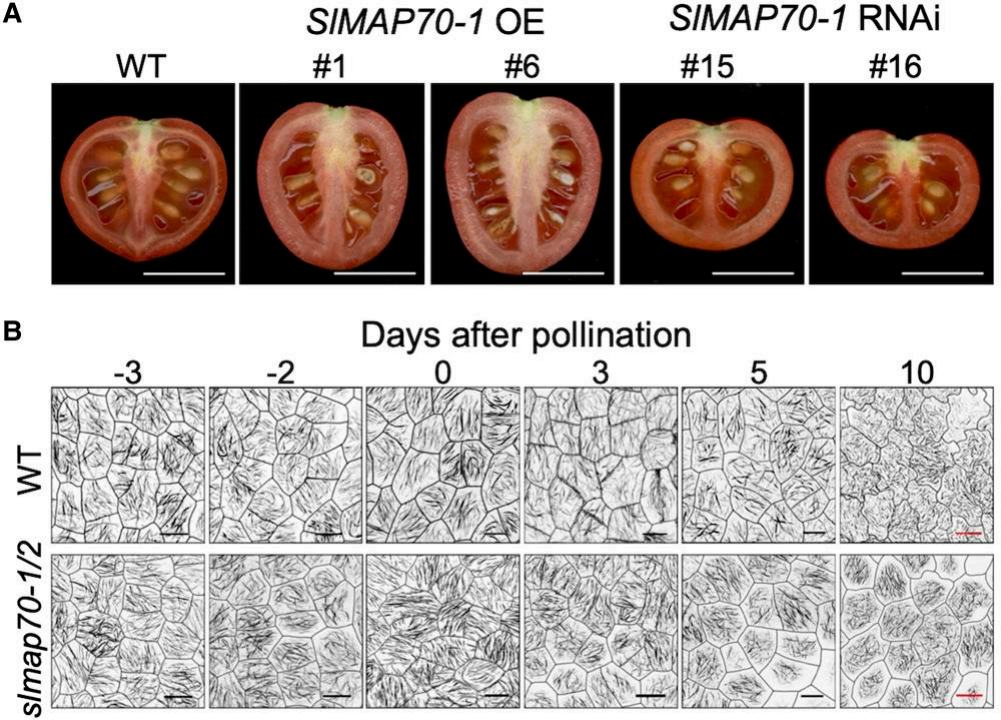
**A)** *SlMAP70-1* expression affects tomato fruit shape. **B)** Cortical microtubule arrangement (GFP-MAP65-1 marker line) in tomato fruit cells during fruit growth in the WT and *slmap70-1/2* mutant. Scale bars represent 1 cm in panel A. Black bars: 10 *μ*m; red bars: 25 *μ*m in panel B. OE, overexpressing lines; WT, wild-type. Adapted from Bao et al. (2023), Figures 2 and 4.

The authors used live-cell imaging in the endocarp (a fruit tissue) to track the microtubule organization and dynamics. Anisotropy values (reflecting order in the microtubule filaments) in endocarp cells were similar from −3 to 0 days after pollination; after that, a dramatic re-orientation of the microtubules occurred. However, in the *slmap70-1/2* double mutant, this re-arrangement was affected and the order in the microtubule filaments remained similar until anthesis and after pollination. Thus, microtubule re-arrangement mediated by the SlMAP70 proteins at early stages may be crucial for organizing cell morphogenesis and the final fruit shape.

To identify other microtubule-related proteins controlling fruit shape, a yeast 2-hybrid assay was performed using SlMAP70-1 as bait; SlIQD21a (SUN10) was identified as a putative interaction partner. The IQD family has 33 members in tomato and several were selected to generate overexpression lines. The overexpression of *SlIQD1* generated extremely elongated fruits; the effect of *SlIQD21a* was similar to *SlMAP70-1*, suggesting a cooperative function. The combined overexpression of *SlMAP70-1* and *SlIQD21a* further enhanced fruit elongation. Analysis of cell features in these additional lines indicated that anisotropy in microtubules increased in the mutant line *slmap70-1/2* and was reduced in the *SlMAP70-1* overexpression line. Long fruits (overexpression of *SlMAP70-1* or *SlIQD21a*) correlated with reduced cell circularity. In *Arabidopsis* pavement cells, high circularity correlates with low microtubule anisotropy, and cells expand isotropically. Interestingly, the opposite was observed in tomato, where increased microtubule anisotropy in endocarp cells was correlated with high circularity.

Microtubule dynamics were also analyzed at the tissue level to document the correlation between microtubule patterns, cell shape, and fruit shape. Endocarp cells in the *map701/2* double mutant displayed no preference for microtubule direction with respect to the growth direction, but lines overexpressing *SlMAP70-1*, *SlIQD21a*, or both showed an enrichment in transverse microtubules. Microtubules were more ordered in the *slmap70-1/2* at the single cell level, but the average orientation varies from cell to cell so that cells can expand in different directions; on the other hand, in the overexpression lines, microtubules were oriented transversely, restricting lateral expansion and favoring cell elongation, resulting in elongated fruits.

With respect to microtubule organization, the overexpression of *SlIQD21a*, *SlMAP70-1*, or both induced the formation of thick microtubule whorls and bundles, while this phenomenon was reduced in the double-mutant *slmap70-1/2*. Microtubule dynamics were also affected; in the wild-type, microtubule polymerization and depolymerization occurred at similar rates; however, in plants overexpressing *SlMAP70-1* or *SlIQD21a*, microtubules were more static. The function of *SlMAP70-1* and *SlIQD21a* as microtubule stabilizers was measured after chemical treatments. High concentrations of oryzalin disrupted the microtubules after 10 min; however, the increased expression of *SlMAP70-1* and *SlIQD21a* induced a protective effect, and intact microtubules were detected 40 min after treatment.

In summary, the SlMAP70 proteins and SlIQD21a contribute to microtubule stability, affecting microtubule rearrangements and cell growth patterns. This work highlights the importance of microtubule organization and dynamics in defining organ shape.

## Dive Curated Terms

The following phenotypic, genotypic, and functional terms are of significance to the work described in this paper:

locule AmiGo: PO:0025263

endocarp AmiGo: PO:0009086

fruit AmiGo: PO:0009001

fruit shape Planteome: TO:0002628

SlMAP70-1 Gramene: Solyc05g017980
SlMAP70-1 Araport: Solyc05g017980

SlIQD21a Gramene: Solyc03g083100
SlIQD21a Araport: Solyc03g083100

SUN1 Gramene: Solyc10g079240
SUN1 Araport: Solyc10g079240
