# Correction to: Not another round: Microtubule-associated proteins and the control of fruit shape in tomato

**DOI:** 10.1093/plcell/koad287

**Published:** 2023-12-14

This is a correction to: Humberto Herrera-Ubaldo, Not another round: microtubule-associated proteins and the control of fruit shape in tomato, *The Plant Cell*, 2023; https://doi.org/10.1093/plcell/koad238

In the originally published version of this manuscript, there was a lack of distinguishing formatting between the names of proteins and genes. For genes, formatting is corrected to read: “*SlMAP70*” and “*SlMAP70-1*” instead of: “*slmap70*” and “*slmap70-1*”; and for proteins it has been corrected to read: “SlMAP70” and “SlMAP70-1” instead of: “*slmap70*” and “*slmap70-1*”.

This point of formatting has been emended in the article.

